# When ADHD knocks on the door – discourse theory as a frame to explore subject positions and mental wellbeing before diagnosis

**DOI:** 10.1080/17482631.2023.2209964

**Published:** 2023-05-08

**Authors:** Siv Vea Grønneberg, Eivind Engebretsen, Stine Torp Løkkeberg

**Affiliations:** a Faculty of Health, Welfare and Organisation, Østfold University College, Fredrikstad, Norway; b Institute of Health and Society, University of Oslo, Oslo, Norway

**Keywords:** ADHD, discourse analysis, subject positions, emotional needs, societal norms, wellbeing

## Abstract

**Purpose:**

Attention deficit hyperactivity disorder (ADHD) is currently the most prevalent childhood psychiatric diagnosis. This article reports how 10 young adults in Norway positioned themselves before they were diagnosed with ADHD either during early childhood or adolescence. A central theme is how these subject-positions relate to societal norms and mental well-being.

**Method:**

Individual interviews were conducted, and the transcriptions of the interviews were analysed according to discourse theory.

**Result:**

Six central subject positions were identified which in turn related to two major positions: 1) failure with schoolwork and 2) struggle in social interaction. The findings indicated specific emotional and environmental needs and that individuals were confronted with societal norms related to the criteria for ADHD prior to and regardless of whether one had received a formal diagnosis.

**Conclusion:**

We argue that analysing subject positions provides important knowledge regarding ADHD that is useful for individuals, parents, teachers, practitioners, policymakers, and helping professionals in the field of mental health and education when it comes to interventions that support children who possess different temperaments.

## Introduction

An increasing number of children and adolescents are diagnosed with attention deficit hyperactivity disorder (ADHD) (Polanczyk et al., 2014). In Norway, 3–5% under the age of 18 are diagnosed, which means there is on average one child with ADHD in each school class (Ørstavik et al., 2016). This increasing number of children being diagnosed with ADHD indicates an increasing call for diagnostic assessments from healthcare (Atladottir et al., 2015; Bor et al., 2014; Dalsgaard et al., 2013).

According to the Diagnostic and Statistical Manual of Mental Disorders (DSM), the leading classification manual in the Western world, ADHD indicates difficulties in relation to three core symptoms: 1) failure to sustain attention and concentration, 2) reduced impulse control, and 3) hyperactivity/overactivity and agitation (American Psychiatric Association, 2013). Symptoms that target both social interaction and cognitive function, had an impact on individuals’ self-understanding and identity, which can be a considerable personal and individual burden (Katzman et al., 2017; Thoits, 2012).

The criteria for the diagnosis (in the DSM) are based definitions of behaviours that are considered as “wrong” and unwanted, e.g., “doesn’t do as told”, “doesn’t sit still”, “disturbing others”, etc.. Such focus causes the understanding of the emotional level in children, their connection to other people, and to the contexts around them often goes missing (Timimi, 2017). Timimi and Leo (2009) emphasized that classification based on criteria of what is “wrong” can be helpful in validating suffering and for accessing resources but is of little use to explain experiences, behaviours, and which approach might be most useful.

Discourses that are related to behaviour contain norms and expectations set by the public and political systems, in which highly reputable systems (such as the DSM) hold power relations that construct positions for people and influences on how we understand ourselves and are understood by others (Becker, 1967; Foucault, 1971; Winther Jørgensen & Phillips, 1999). Seeing ADHD as a social construct, influenced by societal norms and expectations, provides a non-pathologizing starting point for helping children and their families (Timimi, 2017). In this regard, Harre (2005) argues that exploring subject positions, the individuals own experiences, can be an excellent way to meet someone’s complaining or someone being complained about—and a good alternative to error and omissions-based classifications.

As two out of three diagnosed with ADHD as children and adolescents experience symptoms that persist into adulthood (Ørstavik et al., 2016), exploring subject positions before diagnosis appears essential, as early detection of symptoms prevents developments regarded as negative for mental well-being, as well as for optimizing treatment (Katzman et al., 2017; Sonuga-Barke et al., 2011). Also, ADHD has comorbid symptoms that might be mixed with other behavioural disorders, such as autistic spectrum condition (Magon, 2017), and as many as 80% of the adults with ADHD might have at least one coexisting psychiatric disorder (Sobanski et al., 2007; Torgersen et al., 2006). Another factor for early detection, is that behavioural symptoms noted as ADHD may correspond with symptoms of trauma and stressful life events, and thus be behaviours that are expressions of something challenging in the child’s life (Ford & Connor, 2009; Reigstad & Kvernmo, 2015).

In the search for the literature, there were hardly any empirical studies regarding approaches to ADHD or other behavioural diagnosis that used discourse theory to explore experienced subject positions and needs in the context of the child’s environment. In fact, Tuukkanen and Pekkarinen (2022, p. 1) claim that “no theoretical framework has been developed from the perspective of the state of the environment”, which called for more knowledge of ADHD in relation to norms and societal structures.

A study of adolescents’ experiences of being diagnosed with ADHD described struggles with vulnerability, with responding to a label and with manoeuvring in social life. Painful disappointments regarding support and treatment effects were also noted, which suggested that the consequences of the diagnosis needed more recognition and needed to be reflected before diagnosis, as well as when conveyed to and in the follow-up (Andersson Frondelius et al., 2019).

A meta-synthesis review of qualitative research on living with ADHD highlighted that behaviours, thoughts, and emotions regarded as unwanted were related to their diagnosis, rather than to their own will. Also that their identification with the disorder was shaped by their ambivalences towards their psychological needs and the closeness of their social environment (Ringer, 2020).

Experiences of interpersonal conflict, stigma, and invalidation that had a negative psychological impact on self-esteem and identity were emphasized in a systematic review of adolescents living with ADHD. Findings suggested that support strategies were needed to support resilience, the individuals abilities and autonomy, and called for more qualitative research regarding the transition of young people’s identities to ensure what clinical practice is most helpful for their psychological well-being (Eccleston et al., 2019).

Previous research has also highlighted the need for clinicians, educators, and parents to be more responsive to the needs of children with ADHD, as there might be “a gap between how those with ADHD understand their experience and how their clinicians seem to understand their experiences” (Miller, 2017, p. 44). As different people have different symptoms, a qualitative approach that favours understanding individual’s own histories can be significant to get into the origins of a particular social phenomenon and the subjective reality (Rigg & Murphy, 2013) and be the bridge that can describe behavioural antecedents, settings, and consequences (Presser, 2009).

Hence, this study used discourse theory as a frame to explore subject positions and to tap into young adults’ own retrospective perspectives of available subject positions before they were diagnosed, asking Which subject positions that may represent a barrier to well-being, do young adults experience being available before they were diagnosed with ADHD? Which norms and societal structures can be related to such positions, and which emotional and environmental needs do the identified positions refer to and reflect?

The purpose of this article was not to enter into a discussion about what ADHD is, how ADHD originates, or what treatments are most effective. The ambition was to expand and deepen the understanding of the phenomenon of ADHD by focusing on the experiences of those living with the diagnosis. In this regard, to enable individuals with symptoms related to ADHD and for, e.g.,, teachers, parents, caregivers, and politicians, to better understand how salient behaviours and emotional needs can relate to specific norms and societal structures. Rather than focusing on the limitations of the individual, the method focused on the limitations of the available subject positions that the individual was assigned by society.

## Methodology

### Theory and method

The method used in this study was inspired by discourse analysis and analysis of subject positions (i.e., children and adolescents). Winther Jørgensen and Phillips (1999) point out that discourse analysis, with its social constructionist foundation, is both a theory and a method, as it is based on the theoretical assumption that language shapes social reality for people. In this way discourse theory provides a framework for analysing the ways in which language and communication construct a social reality that includes subject positions. Foucault (1971) argues that subjects are created in discourse by identifying with specific ways of being and distancing ourselves from others. This is a process that is not only controlled by the individual but through interpellation. Althusser (1999) explained interpellation as a process in which language contributes to constructing a social position of the individual. Laclau and Mouffe (2001) used the term articulating about the way phenomena become linguistic, which in turn contributes to the construction of self-understanding and identity.

Discourse is also about what is not said—as one truth may exclude and other truth, and in this regard point to another truth (Winther Jørgensen & Phillips, 1999). Foucault (1971) used the term exclusion mechanisms when highlighting that new ways of thinking can emerge by finding what a discourse excludes. In this regard, antagonism, the discourse theory’s concept of conflict, emphasizes that an antagonistic relationship occurs between positions when, e.g., a position considered negatively harmful for mental well-being stands in the way of, or excludes, a position that appears more appropriate (Winther Jørgensen & Phillips, 1999).

In finding linguistic patterns during discourses, and in the “pursuit” of subject positions, there is a need to account for what is implied in the statements (Boréus & Bergström, 2017). For example, being “someone who has difficulty navigating in life”, could be an image representing the difficulties in moving forwards within an environment that appears chaotic and confusing.

In sum, one thinks that statements are performative in that they contribute to creating a reality for people based on the positions the discourse offers. One consequence is that discourses can control who is “within”, who is “outside”, and who becomes the psychiatric patient (Winther Jørgensen & Phillips, 1999). In other words, discourses offer “certain ways of seeing the world and certain ways of being in the world” (Davies & Harré, 1999, p. 35).

With this study, discourse theory provided a lens that allowed focusing on “how a person negotiates who to be within certain discursive terrains” (Jansen & Andenæs, 2013, p. 122). The research questions were as follows: Which subject positions that represented a barrier to mental well-being, did young adults experience was available before they were diagnosed with ADHD? Which norms and societal structures can be related to such positions, and which emotional and environmental needs do the identified positions refer to and reflect?

### Selection

The study is based on 10 individual interviews with five men and five women, aged from 19 to 28 and diagnosed when they were between the ages of 6 and 17 years old. The mean age was 22.3 years. The age group was chosen based on the close proximity in time from being children and adolescents, and at the same time in a period of their lives where they were distant enough from the time period in reflection. The average age when the interviews took place was 22, the average age when diagnosed was 10 and thus was on average 10 years since they were diagnosed.

The selection was strategic in that there were guidelines in relation to age and equal distribution of gender. There was no intention to exclude informants with “double diagnoses”. Inquiries with information about the project were sent to three high-school counsellors and one counsellor in “advice and health” at a college. These were employees who, through their work and network, had contact with young people with ADHD and who initiated contact with the relevant candidates.

Potential participants were sent a letter inviting them to participate, which also described informed consent procedures. A consent and information sheet was sent to the participants, and they had to sign in order to participate in the study. The selection process occurred continuously and in parallel with the ongoing data collection to achieve the desired variation. This continued until we concluded that the breadth was deemed acceptable and that the material had sufficient informational strength to answer the question. Those who contributed to the recruitment were then informed that the project had enough participants.

Those that responded favourably were interviewed, and 10 individual semi-structured interviews were conducted with room for leaps of thought and digression. The questions were aimed at experiences of living with a diagnosis in general. However, experiences about the time before diagnosis came up as something that grew up organically from the conversations, which inspired this article. Table I shows samples of the initial questions from the interview guide.

**Table I.** Sample of initial interview questions.

How did you experience receiving your diagnosis? (The feeling when you got the message) Did this change anything – how? Before you were diagnosed, did you experience difficulties in your everyday life, or did others bring this up? How did you learn about the diagnosis? Have you tried to acquire information about the diagnosis yourself? Is it important for you to understand what causes ADHD? What does the diagnosis mean for you?

The interviews were conducted by one researcher in suitable places near where the individual resided or in the individuals’ homes, while three interviews were conducted digitally through Zoom. They lasted 35–70 minutes, with an average time of 55 minutes and were audio recorded and transcribed directly after completion. The participants appeared to be similar in the sense that they had the same ethnic background, were mainly from the same economic class and the same part of Norway. Three also experienced reading difficulties and one dyscalculia. One of the participants was also examined for diagnosis within the autism spectrum.

Since the participants in this study had completed upper secondary school, were in or had completed vocational education and were students (one has completed a bachelor’s degree), they all had a certain success in schooling. However, despite such success, all had experienced positions with significant limitations for their wellbeing.

### Ethics

To meet ethical issues, all names in this study are fictitious and other details, which could lead to the identification of the participants have been changed or omitted. Informed consent was obtained from all individual participants included in the study. The study was approved by the Research Ethics Committee (REC) (ref: 2019/85) and by the Norwegian Centre for Research Data (ref: 216926). Data are contained in TSD (Service for Sensitive Data).

### Analysis

To identify subject positions that were experienced available before diagnosis, all transcriptions were read and reread very carefully. Then, as the first step of the analysis, a case was created for each participant within the NVivo 12 computer programme, where all the interview transcriptions were conducted.

To operationalize the analysis, we leaned on Laclau and Mouffe’s (2001) theory of core concepts. The core concepts became vital because they were associated with and defined by a series of other concepts that were part of the so-called chains of equivalence. Each of such chains of equivalence belongs to a central point or master signifier (Winther Jørgensen & Phillips, 1999). For example, expressions, such as “one who always gets blamed” and “one who always gets yelled at”, became part expressions of a chain that pointed to a central master signifier (subject position) as “scapegoat”.

First, all expressions related to barriers for wellbeing were identified and collected in a folder. These expressions typically referred to characteristics, actions, feelings, and conditions that could be regarded as unwanted. Those expressions that are related to each other were put together and collected in the corresponding subfolders, which in turn made up six chains of equivalence that pointed to six central master signifiers (subject positions). When we studied the six chains and their corresponding subject positions, it became clear that these again consolidated into two main positions: 1) school-related struggles and 2) struggles in social relations.

We then focused on statements that referred to conditions and feelings. These were those articulated directly, such as “I felt stupid” or “I became more withdrawal”, but also those that were implied in the statements (Boréus & Bergström, 2012), e.g., “I had much trouble in navigating”, which indicated experiences of chaos and helplessness.

Further, when we aimed to identify emotional needs, we leaned on Foucault’s (1971) principles of exclusion mechanism and antagonism and for what was implied in the statements (Boréus & Bergström, 2017). For example, references to the animal kingdom, such as “I was the black sheep”, indicated a position of lower value (i.e., antagonist) and in conflict with a position as “one who is valuable” which in turn pointed to the need to feel “valuable”.

The same principles were used when we aimed to identify norms and societal structures that are related to the identified positions. For example, experiences in the face of school, such as of “being overstretched”, “feeling stupid”, and of “having to perform beyond one’s abilities”, questioned society’s requirements for equal school maturation and pointed to the need for a school that is more adapted to the child’s abilities and a need for arenas for experiences of coping.

Relevant quotes from each participant were marked and drawn in folders corresponding to the various positions. In addition, the programme kept track of what was said by the specific participants. In this way, each participant also became “visible” for the analysis with their unique experiences and positions and descriptions “offered”.

## Results

From the expressions in the interviews, six central positions with six corresponding chains of equivalence were identified. These six positions gathered under two major positions: (1) struggles with schoolwork and (2) struggles with social interaction.

The classification of these identified positions cannot be regarded as absolute—struggle regarding schoolwork had consequences for the social and relational and vice versa. There are also variations within each position; the participants can hold several positions simultaneously. Therefore, there is no defined dividing line between the two main positions. Table II shows six chains of equivalence based on expressions used by participants, and the subject positions for which these chains correlated.

**Table II.** Six chains of equivalence and the central and major positions they are related to.

Equivalence chains	Subject positions	
dyscalculia, math difficulties, reading and writing difficulties, stupid at school, learning difficulties, struggling with subjects, someone who does not understand, misunderstands, stupid, someone who gets much help from mother	In need of help	Struggle with schoolwork —- Struggle in social inter-action
inattentive, have difficulty concentrating, inside hyperactive, spaces out, distant, daydreams, scatterbrain, has attention, deficit, has thought chaos, forgets things, absent-minded, loses things, easily confused, has difficulty navigating life, under-performs, someone who forgets agreements, has a maelstrom of thoughts	Chaos and lack of perspective	
restless, hyperactive, cannot sit down, can’t sit still, runs around school, has verbal diarrhoea, talks without stopping, uncontrolled, lacks impulse control, explosively angry, class clown, spaces out, has thoughts that drift	Restless and lack of control	
outcast, different, weird, defective, weak, someone that is not seen and heard, introvert, makes animal noises, one who needs to adapt, putted in a box, one who has extra luggage, comes too late, one who oversleeps and is late, invisible, the strange one, introvert, someone that arouses concern,	Not being as expected	
condescending, sarcastic, talks to people like they are trash, argumentative, is mad, communication difficulties, annoying, has anger issues, pathological liar, aggressive, rude, unpopular, stubborn, interrupts others	Arguing and conflict	
someone who always gets blamed, always yelled at, the black sheep, has a personality fault	Scapegoat	

In the following, we will provide a more detailed description of how these positions expressed the conditions and emotional needs associated with the subject positions, and how these positions reflected the environment they operated within.

**1) Six positions that related to failure with schoolwork and struggle in social interaction**

**In need of help**. The school appears to be an arena where the positions of “in need of help” and lack of mastery are particularly evident. Most say, they have experienced defeat, felt stupid, as a failure, and much pressure to perform academically. Most experienced the feeling of being “overstretched” and fear of “standing out”.

Essentially, we found no differences in responses that directly could be related to gender regarding the time before diagnosis; however, the fear of being “left behind” with schoolwork were more expressed by the girls. For example, Nina says she was a “good girl” and spends a lot of (too much) time on homework. Inherent in being a “good girl” is an image of being dutiful and going to great lengths not to make a mistake: “(…) I spent a lot of time on homework … could sit there doing homework for three hours … I had to … I wanted to be on an equal footing with the others … did not want to fall behind (…)”. The fear of standing out was attributed to feelings, such as shame and fear of stigma – “one who do not master”, “one who do not complete tasks and subjects”, and “one who are different from others”. In this regard, positions attributed to the norm of ADHD also appeared as something one does not want to be associated with—something one distances oneself from. In sum, the position “in need of help” reflected the society’s expectations of equal maturity among pupils and that not adhering to the norm is regarded as an aberration and disability.

**Chaos and lack of perspective**. Positions, such as being “someone who is inattentive”, a “scatterbrain”, and “someone distant” are much associated with difficulty keeping up with schoolwork, but also a considered part of “everyday life” activities, such as to keep track of appointments and getting up and arriving at school on time. Difficulties in dealing with situations and feelings when confronting one’s environment are highlighted by the *implicit* nature of metaphors, such as having difficulty “navigating through life” – a metaphor that relates to difficulties moving forward in waters that appear chaotic and confusing. Hilde, even though she is good at school, struggles with feelings of chaos and a lack of structure: “(…) it was kind of painful not to understand why I was like that … have always noticed that there was something different … noticed that I have not quite managed to navigate … yes … life and things like that”. The lack of perspective is substantiated by the fact that you do not understand why this is happening to you and that this can be experienced as painful.

**Restlessness and lack of control**. Restlessness manifests itself as internal and external restlessness, where internal restlessness and lack of concentration contributed to difficulties with schoolwork. One participant described inner restlessness as having *a maelstrom* in the head. A maelstrom is implicitly something that can catch you, take control of you, and can drag you down into the undertow—as uncontrolled as nature can be: “There was this maelstrom of thoughts spinning and turning—a thought that flows by and then a new one that comes along … and experiences and stuff do not stick … everything gets mixed up”.

The “class clown” is a term that many participants use about themselves when talking about external restlessness or hyperactivity. Clown is descriptive of someone who is a game maker, invents and does many strange things, but also someone who behaves not as expected and does many stupid things. Per described his role as a class clown as a degrading role: “(…) it was how I coped socially … came up with some pranks … was playful … did some strange things … and got a response (…) it was a feeling of being recognized … despite the degrading role that class clown really is”. It seems worth noting that the behaviour they experience as undesirable by their environment, is also behaviour they distance themselves from and do not appear as intentions of being stubborn or difficult. Behaviour that can be regarded as undesirable appears throughout as a way of dealing with chaos, stress. and frustration, and striving for acceptance and recognition.

**Arguments and conflicts**. Several participants said that there was a lot of being yelled at and that they often argued, especially with parents and siblings. Nina stated that her home conditions were never looked at “I have had an unstable home with a sick mother and conflicts, and in a way unsafe in that sense. If these conditions had been looked more into, my situation might have been different (…)”.

Positions became visible with characteristics and actions that can be associated with conflicts, such as “being a pathological liar”, “one with communication difficulties”, and “one who is annoying”. The difficulties are substantiated by the fact that they do not always understand why they get yelled at. Cecile says “(…) I was yelled at every single day because I did not have any more gloves left each week, I kept leaving them behind and going out … a lot of yelling and arguing characterized my childhood and that I did not understand why I was being yelled at … it was very stressful”.

Positions, such as being “unpopular at school”, also indicate relational difficulties with classmates and the experience of exclusion and “being different”. Per felt that the behaviour gave him a stigma that stuck with him for many years. He understands this as judgement from the environment, which is from fellow students: “(…) I had a foundation from elementary school that I was not very happy with … I stood out because I was the strange one. Was a bit out of control and stuff. And it stuck (when I started middle school) you know … the merciless”.

Per tells this in a way that indicated the experience of offence and disappointment. Implicitly, “the merciless” refers to something that can represent something absolute or decided or something deterministic, something you cannot get away with and something that highlights emotions such as frustration, disappointment, and powerlessness. In powerlessness, there is also passivity. Frustration and powerlessness are highlighted due to not knowing how to handle situations and as a result of the environment's reactions to their behaviour.

**Not as expected**. All participants, both in terms of educational and relational struggle, experienced that they were the ones that had to adapt to their environment and not the other way around. Several stated that they had a strong experience of adjusting, which for some contributed to withdrawal. For example, Stian felt “as normal” before he was diagnosed. “Normal” for Stian meant “just fine”, a position that was nice to live with “It was a teacher who noticed it. I struggled a lot with my thoughts drifting, but I never thought about it … just that I was 7 years old and that it was just fine … but obviously it was not … then I became more withdrawn”.

Experiences of not being as expected are often expressed with references from nature and the animal kingdom, such as “I had a maelstrom in my head”, “I was the black sheep”, “I was an outcast”, “I ran around making animal noises” and so on. The positions were told in a way that confirmed that this was behaviour the environment regarded as undesirable or unacceptable. The animal references were also expressed as positions that were of less or lower value. In this, we also find implicit subject positions, such as “one that is not good enough” and “one that must be fixed” to fit the norm for what is expected. It is apparent in the face of the expectations from the environment, and especially to expectations at school that a position such as “not as expected” stand in the way of a role of position as “one who is valuable” or “good enough. In this way, positions also say something about a norm that dictates that, one must achieve more than the person they are and where there is no room to “be oneself”.

**Scapegoat and self-stigma**. Most of the participants have felt condemnation from their environment in that they often got the blame and yelled at if something went missing or got broken. Karoline: “I was always blamed if something disappeared, then it was somehow my fault—if something had happened then it was my fault”.

It seems worth noting that most participants see themselves as the cause of the difficulties they experience and the subject position as a “scapegoat” and being “someone with personality defects” contributes to feelings of guilt and shame. In this way, the role of scapegoat appears unsuitable for self-understanding. Some also referred to a condemnation of themselves that appeared very harsh and was close to self-loathing. For example, Cecilie asked herself to be assessed in junior high school stated, among other things: “(…) I was very difficult, incredibly aggressive, explosively angry and talked to people as if they were trash, very condescending and sarcastic (…) stubborn is sort of the word for my upbringing”. This was said in a way that indicated the experience of shame and self-stigma. Experiences of having to adapt to environmental demands and to control aspects of one's own situation pointed to specific needs that will be explained in more detail under the next point.

**2) Emotional and environmental needs**

The mentioned positions, feelings, and conditions appeared in an antagonistic relation to specific positions that referred to and reflected specific emotional and environmental needs (Table III).

**Table III.** An overview of the positions, conditions, and emotional needs, which the central subject positions are reflected and referred to.

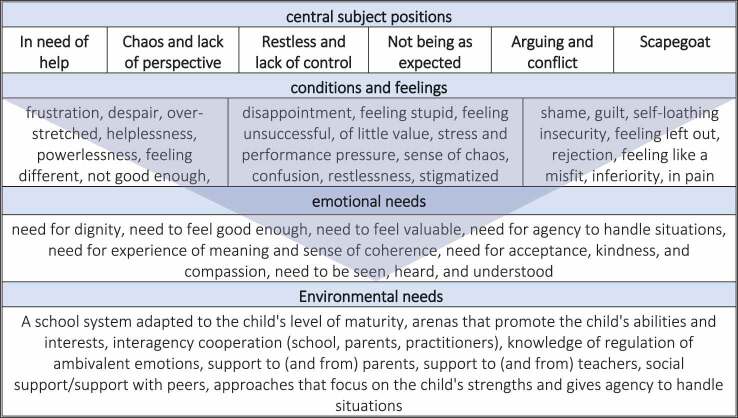

Most of the central positions were experienced as “not being good enough”, and as Per said; “a degrading role”. Antagonism aroused when such a position excluded or stood in the way of a position that could be considered more appropriate for well-being, which in turn reflected specific needs and conditions to obtain a position as “good enough”. These needs will be reflected and presented in the following (Table IV).

**Table IV.** An example of antagonism between positions and of individual and environmental needs that a position as “not good enough” reflected.

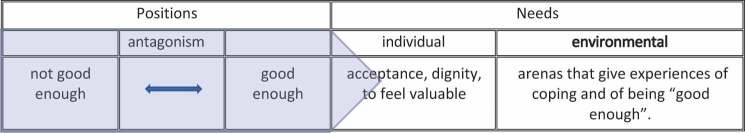

*The need for dignity, to feel valuable and good enough* were highlighted by statements, such as “I was an outcast” and by references to the animal kingdom, such as “I was the black sheep”. The aforementioned positions and emotional needs framed certain societal norms and expectations, especially expectations in the face of school contributed to experiences of positions of lower value. These statements were portrayed in ways that indicated feelings and positions of being “one who has lower value that others” and “not good enough”, which stood in an antagonistic relationship that excluded, a more appropriate position such as “one who is valuable” and “good enough”. Experiences of failure at school and roles, such as the “class clown” and of conflicts at home indicated the need for support regarding teachers’ and parents’ knowledge with the regulation of ambivalent emotions. Furthermore, experience of failure at different arenas (school, home, with peers) indicated need for a collaborating enterprise (e.g., practitioners, school, and parents), concerning the child’s needs. Experiences of failure and positions like “in need of help” and “not good enough” pointed to the need for a more adapted school system to accommodate for experiences of failure as well as approaches that focuses on the child’s strengths—arenas where one could achieve coping and could use one’s interests and skills and being “good enough”.

*The need for agency to handle situations, meaning and sense of coherence* were derived from the positions, such as “one with chaos and lack of perspective”, which referred to feelings and conditions, such as frustration, powerlessness, helplessness, and lack of tools to deal with situations. Being unable to master or deal with situations indicated positions of passivity—that one felt stuck and could do nothing about a situation, which pointed to a need for knowledge and approaches that give the child the tools and agency to handle the situations and challenges in one’s life.

*The need for acceptance, kindness, and compassion to be seen, heard, and understood* was much derived from experiences of failure and conflicts at school, arguing with parents, siblings and withdrawal from peers. “The scapegoat-position”, that one sees oneself as the cause of difficulties and loss of self- compassion, shed light on an awareness of how children and adolescent with behaviours regarded as unwanted a met by their environment. Furthermore, arguing and conflicts at several arenas pointed to an awareness regarding parent’s need for support, teachers need for support and for social support regarding peers.

## Discussion

This study aimed to use discourse theory as a framework to identify subject-positions extracted from the transcriptions of individual interviews, which were positions that could represent a barrier to mental well-being. The purpose was also to gain insight into the feelings and conditions the identified positions reflected and referenced, as well as the emotional needs and societal structures these positions reflected.

The analysis revealed six central positions, which were “in need of help”, “chaos and lack of perspective”, “restless and lack of control”, “not being as expected”, “in arguing and conflict” and “scapegoat” - positions that could be regarded as “negative” for self-understanding and mental wellbeing. These positions consolidated into two major positions that were struggles related to schoolwork and struggles in social settings and were consistent with studies that emphasized children with ADHD who have difficulties and subsequent academic and social learning consequences (Lillevik & Øien, 2014; Slåttøy, 2002).

Pressure in relation to school (academic satisfaction) was apparently influenced both on the social wellbeing at school and the reverse. This reciprocation of academic performance and social wellbeing at school reinforced feelings such as chaos, insecurity, frustration, and helplessness. Pingault et al. (2011) highlighted similar findings in that restlessness and lack of control over one’s behaviour and conditions had consequences for academic learning, which in turn had consequences for social relationships, as social difficulties again go beyond the academic difficulties.

The identified positions reflected and referred to certain conditions and feelings that appeared as a barrier or stood in the way of wellbeing. The fact that most saw themselves as the cause of their difficulties, and only blamed their environment to a small extent pointed to a social process that indicated interpellation of positions that influenced self-understanding and identity negatively (Althusser, 1999). Some condemned themselves with harsh positions, which also appeared close to self-loathing, which aligned with studies that emphasized that children and young people can position themselves in ways that can be regarded as undesirable and even unbearable (Davies & Harré, 1990).

The findings also reflected environmental norms and expectations related to ADHD that existed regardless of whether one had received the diagnosis, which also pointed to specific needs for environmental adjustments. Especially, the importance for the child’s environment to be aware of that behaviour considered unwanted might be a sign of distress and adverse circumstances, and a way of dealing with emotional difficulties, such as insecurity, chaos, stress, and confusion and in the absence of more appropriate “tools” to handle their challenges (Hertz, 2008; te Meerman et al., 2017; Timimi & Taylor 2004). These findings will be discussed further in the following.

### Antagonistic relationship pointed to certain feelings, conditions, and emotional needs

Antagonistic relationships were especially highlighted where the participants articulated a self-understanding where they experienced themselves as different from what was expected. In this regard, a position, such as “not as expected” indicated a transfer of the subject to a minority, to “the others”, which pointed to a social process that created distinctions between a “we and the others” - those who fall outside and no longer belong to the “we”, those who are vulnerable and in need of help (Engebretsen & Haldar, 2010, p. 209).

Søndergaard (2012), emphasized that the experience of not being good enough, indicated conditions that could be attributed to the fight and wish of being like “the others” - someone who blends in as well as the need for dignity and to feel valuable. Also, positions portrayed as being of lower value, “not good enough”, appeared to be an underlying factor in withdrawing from schoolmates and consistent with Olsvold (2014), emphasizing that withdrawal can be regarded as a way to shield oneself from humiliation.

The need for dignity and to feel valuable were also highlighted in several participants who perceived their behaviour as something they were ashamed of, a feeling that comes forth when we are afraid that we will be devaluated by others (Tangney & Leary, 2012). Several also stated that they saw themselves as the cause of their difficulties, rather than blaming their environments. Seeing one-self as the cause of one’s difficulties can lead to sinking further into the problems that one is trying to solve (White, 2007). Shame is also strongly related to self-stigma and in self-loathing, as self-loathing also encompasses the loss of self-love, which may present a risk of various forms of depression, self-harm, and in the most extreme consequence can be the root to suicide (Popovac, 2020). In this regard, findings highlighted the need to be freed from the scapegoat label and to be allowed to see their own identity as separate from the difficulties—not as “truths” embedded in themselves (Hertz, 2008).

Feelings of chaos, helplessness, and powerlessness pointed to the need for different episodes to be linked to perceive meaning—for coping resources and agency to manage one’s own life. These findings aligned with studies that emphasize that mental well-being is essentially related to the fact that what is happening is cognitively understandable and can be explained—that the challenges one faces can be handled with the help of one’s own coping resources or by someone one trusts (Hertz, 2008; White & Epston, 1990). In this regard, findings also highlighted emotional needs that indicated approaches that focus on tools that give the child self-control, attentional skills, coping strategies and hope for enhancing performance in problematic areas (Derryberry, 2002).

### The identified positions indicated need for environmental adjustments

In debates in the professional fields and in the media, stigma is often related to the ADHD diagnosis—that the diagnosis can lead children to perceive themselves as defective and contributes to children developing a negative self-understanding (Brady, 2014; Krueger & Kendall, 2001). However, the identified positions before diagnosis indicated that the norm about ADHD and expectation about whom one should and ought to be, were processes that to a large extent were present before diagnosis was concluded upon—and thus regardless of whether one received a diagnosis.

Several stated that they strongly felt that they had to adapt to the environment and felt much pressure perform “beyond their abilities”, which made them feel “overstretched”. Expectations related to schoolwork pointed to stress and failure that influenced self-image and mental wellbeing and supported research that highlighted that perception of one’s skills and perception of academic achievements were important factors in developing a positive quality of life (Gustafsson et al., 2010; Sommerschild, 1998). Nina spent a lot of energy hiding her problems as she was afraid of “being different” by being left behind academically. This finding was consistent with studies that highlighted that especially girls might hide their problems, which in turn might lead to their difficulties not being discovered (Quinn, 2005, 2008).

Struggles in social interactions, such as arguing and conflicts with parents and siblings, the role as “class clown” and withdrawal from classmates, aligned with studies that emphasized that behaviours and conditions that were regarded as harmful and undesirable appeared to be maintained by situations that occur in interaction social networks and within the family (Henggeler, 2012). In this regard, findings also supported studies that emphasized that behaviour considered to be unwanted was not necessarily because of the disorder itself, but something that resulted from interactions with an environment and requirements that were not sufficiently adapted to the child’s needs (te Meerman et al., 2017).

Catalá-López et al. (2017) highlighted that medication with nervous system stimulants (e.g., methylphenidate) was often used as a treatment alone for ADHD. However, experiences in harsh positions before diagnosis indicated a need for approaches that went beyond medication and that involved the environments, such as teachers and families. In this regard, Krtek et al. (2022), emphasized that the teacher’s knowledge of ADHD determinants, regulation of ambivalent emotions and empathy increased the quality of the teacher-ADHD- student-relationship.

Arguing and conflicts with parents and siblings pointed to an awareness of families' need for support. Nina emphasized that her difficult home-conditions were never looked at, something she thinks could have made her upbringing easier. Previous studies highlighted that families often experienced that they were left on their own with high levels of stress and unmet needs regarding support for their child’s behaviour after being diagnosed with ADHD (Leitch et al., 2019; Olsvold, 2014). In this regard, the findings supported the need for parenting intervention, which in former studies have been highlighted as applicable (Moore et al., 2016). For example, parents who were offered a programme for relational awareness, experienced a successful outcome in that they became more understanding *and* managed to cooperate better with their child (Timimi, 2017).

We found that experiences of failure took place in different contexts and arenas, at school, at home and with peers. LaForge-MacKenzie et al. (2022), emphasized the value of arenas where youths are encouraged to gain positive psychosocial learning and emotional experiences and highlighted participation in sports and extracurricular activities. For example, the importance of artistic activities are highlighted when it comes to arenas that could raise levels of self-confidence, self-esteem, and the sense of belonging (Zarobe & Bungay, 2017). Peer support groups were also found to offer a re-situation from individualized positions of burden to new and vital possibilities (Klein et al., 2019). These studies underscored our findings in the importance of identifying positions that are barriers to wellbeing and in facilitating for arenas where one can feel accepted and “good enough”—arenas that promote interests, abilities, and belonging.

Findings also indicated that expectations of an idealized life as well as the idea of equal maturity among children need to be reflected upon and toned down, not just due to the consequences of the rapid changes and higher expectations in our society, but both children and families could face physical and emotional strains from these expectations (Erlandsson & Punzi, 2017).

In sum, the identified positions framed expectations from the environment, as well as the individuals' need for adjustment, in order to be accepted and “good enough” – which reflected the ambivalence between the need to be accepted as “who you are” and the need for self-adaptation (Ringer, 2020). In line Halldén (2007), findings emphasized that the framework society sets for expected behaviour, for what is “normal”, and how deviations from norms are handled, have consequences for the type of childhood that is accessible.

### Limitations and Strengths

The analysis and discussion were inspired by discourse theory that focuses on how knowledge and truth are generated by power relations embedded in societal norms and discourses. For such, the theoretical framework can be a limitation as it highlights some aspects that might obscure other parts of the material both in interpreting of the meaning extracted from the text and the interpreting the result—after the actual text analysis (Boréus & Bergström, 2012).

The participants were recruited from upper secondary schools and colleges, and in this sense, it could be said to have some degree of success in terms of school performance. They also appeared to be similar in that they were mainly from the same part of the country, had the same ethnic background, much of the same economic class. It would have been an excellent companion to pursue those with ADHD who had different backgrounds as well as to focus on various mental loads and challenges related to, e.g.,, home conditions and comorbidities. However, a fundamental limitation was the qualitative nature of this study, which means there were relatively few participants so one could not simply generalize it to a larger population. There may also be recall bias due to time and memory failure as it is on average 10 years since they were diagnosed.

A strength of the study’s design was that it favoured the individual’s own perspectives and versions of events, which in this regard was the purpose of the study.

### Clinical implications

This study has clinical importance to understand societal structures of power that provide limited positions for mental wellbeing that to a large extent were present before and then regardless of receiving a diagnosis. In such structures, those with difficulties attributed to the criteria for ADHD (and possibly other behavioural diagnoses) would be vulnerable to the feeling of not meeting societal expectations. Frequently experienced positions that indicated a perception of having a lower value, reflected low self-esteem and limited positions in terms of opportunities in life. Findings questioned society’s requirements for equal school maturation and emphasized that exploring subject positions has the potential to add to the individual, parents, teachers, practitioners, and policy-makers an understanding of emotional needs and power relations—how societal structures that are not adapted to the child’s need can be barriers to mental well-being. We argue that exploring subject positions as a method contributed to an awareness of how limited positions are constructed, elevated, and integrated into a behavioural repertoire, and therefore could be an essential contribution to the direction of treatment.

### Implications for further research

To disrupt the maintenance of limited positions, findings indicated a need for an enterprise prior to diagnosis that goes beyond medication alone. The findings pointed to a collaborative enterprise that involves parents, peers, and helping professionals in education and health care. For future directions, the findings pointed to a need for societal structures, especially educational structures, where children and adolescents are given the opportunity for identity construction through which they can reflect and develop. Further research needs to be able to understand gender differences or differences in people with just ADHD versus people with higher mental health needs. Given the experiences of bullying and being blamed, it would be worth doing further qualitative research in schools and develop interventions to stop young people with ADHD struggling with school and feeling alienated.

## Data Availability

Data are contained in TSD (Service for Sensitive Data Norway) and are not available for open access.
